# Towards Multimodal Equipment to Help in the Diagnosis of COVID-19 Using Machine Learning Algorithms

**DOI:** 10.3390/s22124341

**Published:** 2022-06-08

**Authors:** Ana Cecilia Villa-Parra, Ismael Criollo, Carlos Valadão, Leticia Silva, Yves Coelho, Lucas Lampier, Luara Rangel, Garima Sharma, Denis Delisle-Rodríguez, John Calle-Siguencia, Fernando Urgiles-Ortiz, Camilo Díaz, Eliete Caldeira, Sridhar Krishnan, Teodiano Bastos-Filho

**Affiliations:** 1 Biomedical Engineering Research Group—GIIB, Universidad Politécnica Salesiana (UPS), Cuenca 010105, Ecuador; avilla@ups.edu.ec (A.C.V.-P.); ccriolloa0@est.ups.edu.ec (I.C.); jcalle@ups.edu.ec (J.C.-S.); furgiles@ups.edu.ec (F.U.-O.); 2 Department of Electrical Engineering, Universidade Federal do Espírito Santo (UFES), Vitoria 29075-910, Brazil; carlostvaladao@gmail.com (C.V.); araujos.leticia@gmail.com (L.S.); yvesluduvico@gmail.com (Y.C.); lucas.lampier@hotmail.com (L.L.); luarakerlen@hotmail.com (L.R.); delisle05@gmail.com (D.D.-R.); camilo.diaz@ufes.br (C.D.); elietecald@gmail.com (E.C.); 3 Department of Electrical, Computer, and Biomedical Engineering, Toronto Metropolitan University, Toronto, ON M5B 2K3, Canada; garima.sharma@ryerson.ca (G.S.); krishnan@ryerson.ca (S.K.)

**Keywords:** COVID-19, respiratory diseases, telemedicine, diagnosis, biomedical sensors, machine learning

## Abstract

COVID-19 occurs due to infection through respiratory droplets containing the SARS-CoV-2 virus, which are released when someone sneezes, coughs, or talks. The gold-standard exam to detect the virus is Real-Time Polymerase Chain Reaction (RT-PCR); however, this is an expensive test and may require up to 3 days after infection for a reliable result, and if there is high demand, the labs could be overwhelmed, which can cause significant delays in providing results. Biomedical data (oxygen saturation level—SpO2, body temperature, heart rate, and cough) are acquired from individuals and are used to help infer infection by COVID-19, using machine learning algorithms. The goal of this study is to introduce the Integrated Portable Medical Assistant (IPMA), which is a multimodal piece of equipment that can collect biomedical data, such as oxygen saturation level, body temperature, heart rate, and cough sound, and helps infer the diagnosis of COVID-19 through machine learning algorithms. The IPMA has the capacity to store the biomedical data for continuous studies and can be used to infer other respiratory diseases. Quadratic kernel-free non-linear Support Vector Machine (QSVM) and Decision Tree (DT) were applied on three datasets with data of cough, speech, body temperature, heart rate, and SpO2, obtaining an Accuracy rate (ACC) and Area Under the Curve (AUC) of approximately up to 88.0% and 0.85, respectively, as well as an ACC up to 99% and AUC = 0.94, respectively, for COVID-19 infection inference. When applied to the data acquired with the IMPA, these algorithms achieved 100% accuracy. Regarding the easiness of using the equipment, 36 volunteers reported that the IPMA has a high usability, according to results from two metrics used for evaluation: System Usability Scale (SUS) and Post Study System Usability Questionnaire (PSSUQ), with scores of 85.5 and 1.41, respectively. In light of the worldwide needs for smart equipment to help fight the COVID-19 pandemic, this new equipment may help with the screening of COVID-19 through data collected from biomedical signals and cough sounds, as well as the use of machine learning algorithms.

## 1. Introduction

COVID-19 is a well-known contagious infectious disease caused by the new Severe Acute Respiratory Syndrome Coronavirus 2 (SARS-CoV-2) [1]. Since its first detection in 2019, new variants of SARS-CoV-2 have emerged [2], such as the United Kingdom (UK) variant (B.1.1.7), the Brazilian variants (P.1, P.2, and N.9), the South Africa variant (B.1.325) [3,4], omicron (B.1.1.529), firstly detected in Africa, ihu (B.1.640.2), detected in France, the recent hybrid variant deltacron (AY.4/BA.1), which is a combination of the variants delta and omicron, and the newer recombination of variants BA.1 and BA.2 of the omicron variant (named XE), firstly detected in the UK in April 2022. Current official data, from 2 June 2022, provided by the Center for Systems Science and Engineering (CSSE) at John Hopkins University (JHU), shows that COVID-19 has affected more than 531 million people worldwide, killing almost 6.3 million people, including more than 666,800 Brazilians, 41,100 Canadians, and 35,600 Ecuadorians [5].

It is a fact that massive vaccination (reaching more than 90% in some countries) has prevented or attenuated the effects of this infection, strongly decreasing the number of deaths [6]. However, the COVID-19 pandemic is not over yet, as evidenced by recent lockdowns in Shanghai and Beijing. In addition, the average worldwide vaccinated population is only 60% (some countries have vaccinated less than 10% of their populations) [7], and the current number of deaths is more than 3000 people daily [5].

Depending on the SARS-CoV-2 variant, the symptoms of COVID-19 can include fever or chills, cough, shortness of breath or difficulty breathing, headache, muscle or body aches, dizziness or fatigue, sore throat, congestion or runny nose, new loss of smell or taste, nausea, vomiting, diarrhoea, abdominal pain or anorexia, confusion or impaired consciousness, and rash, among others [8]. Currently, according to the Centers for Disease Control and Prevention (CDC), persons infected with the omicron variant, which represents 99.8% of infection worldwide, can present with symptoms similar to previous variants.

These infected persons may be asymptomatic or symptomatic, the latter varying among mild, severe, and critical. There are risk factors that increase the chance of developing the severe and critical version of the disease, such as advanced age, smoking, and comorbidities (diabetes, hypertension, cardiovascular disease, obesity, chronic lung disease, and kidney disease) [8]. Reverse-Transcription Polymerase Chain Reaction (RT-PCR) is the gold-standard to detect SARS-CoV-2 infection [9]; however, its high cost limits access in countries such as Ecuador and Brazil, where this exam costs between USD 45 and 65 (and more than USD 100 in Canada and other countries). In addition, RT-PCR is only more reliable when the sample is obtained up to three days after getting the infection, and if there is a high demand, test results can be delayed by some days.

Due to the high transmission rate of the omicron variant (much higher than the previous ones), specific measures are still needed to reduce the spread of the pandemic, such as alternative diagnostic methods for asymptomatic and symptomatic individual detection using Artificial Intelligence (AI) [10]. It is worth mentioning that about 40 to 45% of individuals with COVID-19 are asymptomatic [11], which is a big concern to prevent the virus’s spread, as such individuals keep transmitting the virus without realizing that they are.

Thus, a very strong effort has also been made by researchers and industries worldwide to develop low-cost wearable devices and user-friendly mobile applications to detect the symptoms of COVID-19 using information from some biomedical signals and markers, such as cough, heart rate variability, blood pressure, body temperature, and oxygen saturation level [10,12,13]. However, these biomedical data are not decisive to confirm infection by COVID-19, but could open avenues to be used as a screening tool for telemedicine or remote monitoring. For instance, although the sound of forced cough is able to provide a COVID-19 diagnosis, such as claimed by [13,14], another study [15] computed that only 59% of people infected by COVID-19 have a dry cough. On the other hand, the heart rate variability is another separate factor able to indicate possible infection by the virus even in asymptomatic people [16,17].

Body temperature is measured to check fever in individuals, which is another symptom that affects 99% of symptomatic individuals with COVID-19, although it does not occur in asymptomatic ones [15]. Another symptom of COVID-19 is the decrease in oxygen saturation level in blood (abbreviated as SpO2—peripheral capillary oxygen saturation), and when it is below 95%, this may cause serious health issues; hence, there is a need to regularly monitor it. However, other respiratory diseases such as cold and flu also reduce the SpO2 level within the range 90–95% without causing any major health concerns.

The use of Artificial Intelligence (AI) based on biomedical data for the diagnosis of respiratory diseases is quite recent. For instance, ref. [18] presented a systematic review of works that address the diagnosis of pneumonia through several biomedical signals (including the most common ones: body temperature, abnormal breathing, and cough) and using different techniques of AI, such as Logistic Regression (LR), Deep Learning (DL), Least Absolute Shrinkage and Selection Operation (LASSO), Random Forest, Classification and Regression Trees (CART), Support Vector Machine (SVM), fuzzy logic, and k-Nearest Neighbour (K-NN), among others. The study found that AI could help to reduce the misdiagnosis of COVID-19, since there is significant overlap in COVID-19’s and other respiratory diseases’ symptoms.

Recent research has been conducted using samples of sounds from individuals to infer infection by COVID-19 [19,20,21,22,23]. For instance, in [22], a public crowdsourced Coswara dataset, consisting of coughing, breathing, sustained vowel phonation, and one to twenty sounds recorded on a smartphone, was used for this purpose. In another study, data from the INTERSPEECH 2021 Computational Paralinguistics (ComPaRe) challenge were used to infer COVID-19, in a binary classification, through coughing sounds and speech using two subsets from the Cambridge COVID-19 Sound database [22]. The first subset is the COVID-19 Cough Sub-Challenge (CCS), which consists of cough sounds from 725 audio recordings, and the second subset is the COVID-19 Speech Sub-Challenge (CSS), with only speech sounds of 893 audio recordings. In another study [21], an analysis of a crowdsourced dataset of respiratory sounds was performed to correctly classify healthy and COVID-19 sounds using 477 handcrafted features, such as Mel Frequency Cepstral Coefficients (MFCCs), zero-crossing, and spectral centroid, among others. In [23], an audio texture analysis was performed on three different signal modalities of COVID-19 sounds (cough, breath, and speech signal), using Local Binary Patterns (LBPs) and Haralick’s method as the feature extraction methods. Unlike cough sounds, another study [24] used biomedical data (body temperature, heart rate, and SpO2), collected from 1085 quarantined healthy and unhealthy individuals, through a wearable device, to infer COVID-19 infection.

In contrast with the aforementioned studies, the all-in-one Integrated Portable Medical Assistant (IPMA) equipment introduced here uses all these measurements taken together from the individual to improve the diagnosis accuracy of a possible infection due to SARS-CoV-2, instead of using isolated measurements. Thus, this work introduces the IPMA, which is a piece of non-invasive, real-time, any-time equipment for large-scale COVID-19 screening that can be used for daily screening of large populations, such as students at school, employees at work, and other people in general public areas, such as parks, public transit, and more. The IPMA uses four bio-markers (cough, heart rate variability, oxygen saturation level, and body temperature) to infer SARS-CoV-2 infection through machine learning algorithms.

It is worth mentioning that some wearable devices (such as smartwatches and fitness bands), as well as mobile apps have been launched to measure cough, heart rate, body temperature, and oxygen saturation levels. Yet, some of these apps and devices lack clinical validity due to their inaccurate measurements without significant correlation with the measurements of certified clinical devices [25]. In fact, these devices are usually sold as approximate information products that show discrete measurements, indicating that their purpose is to be used as preventive alert devices, to “increase users’ awareness of their general well-being” [26], having no clinical validation or medical certification, nor even indicating the possibility of COVID-19 infection (as they do not have embedded algorithms to do that). In addition, such wearable devices present inconsistency in the quality of data acquisition, and there is no standardization for data collection or sensor placement [27]. Unlike these wearable devices and apps, the IPMA houses medically certified devices and takes their measurements in a row to be stored in a database. In addition, as previously mentioned, although some recent studies have used separately, in different research, human sounds [19,20,28,29,30] and some biomedical data [21] to infer infection by COVID-19, in our study, we used all these signals together to be input into machine learning algorithms, in order to gain a higher accuracy rate. Furthermore, we verified the usability of our equipment with volunteers in terms of the device itself, by using the System Usability Scale (SUS), and of the user interface, by using the Post Study System Usability Questionnaire (PSSUQ).

### Goals

There were three main goals in this study. First was the development of multimodal equipment to automatically acquire data from medically certified devices, without opening them, thus keeping their medical certification, using linear actuators to turn them on, as well as cameras and an algorithm based on a VGG16 pre-trained network to read the devices’ displays. Second was the development of machine learning algorithms to help infer COVID-19 infection. Third was the creation of a database with collected biomedical measurements, which can be further used to infer other respiratory diseases.

## 2. Materials and Methods

### 2.1. Hardware

**Figure 1:**
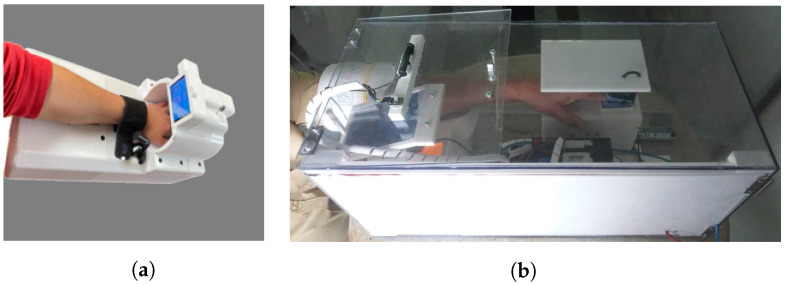
Two versions of the IPMA used in this study. (**a**) Version A. (**b**) Version B.

**Figure 2:**
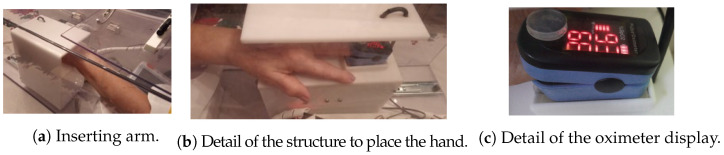
Details of the experiments with the IPMA. In (**a**), an individual inserting his/her arm; in (**b**), the detail of the structure where the hand is placed to measure body temperature and oxygen saturation; in (**c**), a photo of the oximeter display. The upper number (right in the image) is the oxygen saturation, and the bottom number (left in the image) is the heart rate. There is a silicone cover on the power button to avoid the linear actuator damaging the oximeter.

**Figure 3:**
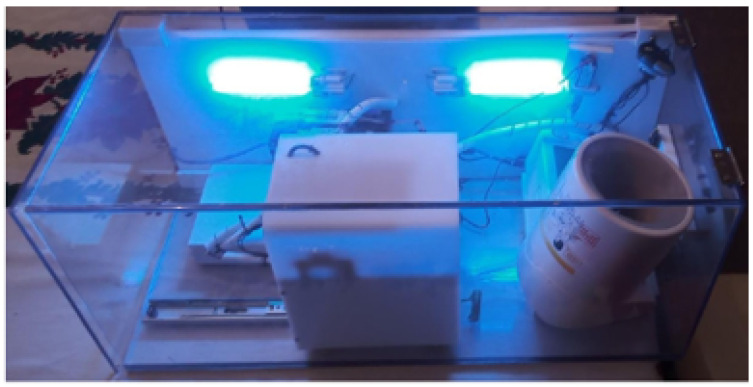
Version B with UV-C applied.

The IPMA used in this study is shown in Figure 1, in two different versions (see the details of these two versions in Table 1 and Figure 2). Inside the IPMA, there is an oximeter, an automatic blood pressure device, a thermometer, and a microphone. These sensors are responsible for acquiring the oxygen saturation levels (SpO2), Heart Rate (HR), Blood Pressure (BP), Body Temperature (T), and cough from the volunteer. Data from the blood pressure device were not used in this study. After every use, the IPMA was completely sterilized, for 2.5 min, using UV-C lamps (see the moment of that sterilization for version B in Figure 3; version A must be put inside a box with UV-C radiation to be sterilized). It is worth mentioning that there was no contact of the volunteer with the UV-C light, which can be harmful to the eyes and skin. In version B, the structure is made of transparent polycarbonate, which blocks the UV-C spectrum. For safety, the UV-C lamps are turned on only if the IPMA’s door is closed, and there is a limit-switch and a control relay that cuts the energy immediately if the IPMA’s door is open, turning off the UV-C lamps.

### 2.2. Software

In order to use the IPMA, a Graphical User Interface (GUI) was developed, which includes a mobile application. The mobile application can store data from the device and send the data to the physician. Furthermore, there is a Flask server that runs a website in an embedded computer, which can be accessed through any browser. On that website, there is a GUI where the user can interact with the IPMA.

## 3. Machine Learning Algorithms

Machine learning algorithms were used here for two purposes: (1) to recognize the characters displayed on the screen using Optical Character Recognition (OCR), from images captured by the cameras; (2) to help the screening of COVID-19 by performing data analysis and classification on the biomedical data captured by the IPMA.

The information about the biomedical data from the individual is acquired by opticam SQ11 mini-cameras focused on the devices’ displays, so that the devices do not need to be opened to obtain that information, thus preserving their medical certification. Once images are acquired, VGG16 is used to adjust the image to a correct position to allow character recognition. The images need to be in the correct position, so that the OCR algorithm can locate and recognize key pixels inside the image to locate the segments of the display and determine whether they are on or off.

### 3.1. Optical Character Recognition

#### 3.1.1. Image Preprocessing

Before applying the OCR algorithm, we performed some image prepossessing steps to segment and standardize the position of the 7-segment display of each image. As the thermometer is well fixed in the IPMA hardware structure, in the preprocessing stage, the image is rotated and cropped according to the region of interest. However, the oximeter needs a more complex preprocessing, as it is attached to a moving structure that adjusts itself to the arm length, and the opening angle of the device also changes the device’s display position relative to the camera, requiring an adjusting algorithm to handle these changes. For this task, we performed transfer learningusing a pre-trained network called VGG16. VGG stands for Visual Geometry Group and is a standard deep Convolutional Neural Network (CNN) architecture with multiple layers. “Deep” refers to the number of layers, with VGG-16 consisting of 16 convolutional layers. It was proposed in [22] to classify images into 1000 different categories. The model with the original weights was provided by the Keras API [23].

**Figure 4:**
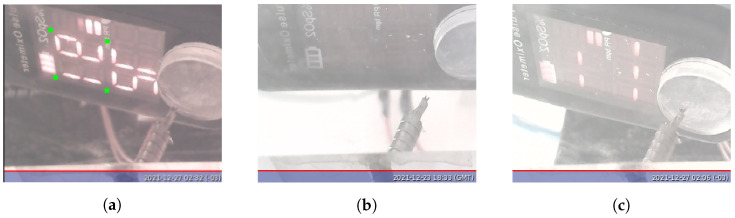
Possible states of the oximeter display. (**a**) Complete reading procedure and points (in green) needed for the oximeter image alignment; (**b**) oximeter turned off; (**c**) incomplete reading procedure.

We adapted VGG16 to find specific points in the oximeter image and used these points to adjust the image for the OCR algorithm. These points are presented in green in Figure 4. To adapt the model, we created a dataset composed of 22 images taken from the oximeter with the position of the marks defined manually. Fifteen out of them are images of the oximeter displaying SpO2 values, and 7 out of them are with the display turned off or for incomplete measurement, as shown in Figure 4.

The input and outputs layers of the VGG16 were modified to our needs. The input layer was swapped for one with the shape of our images (288 × 352 × 3—high × width × colour channels), and the original output was changed to a dense layer with 1024 neurons with the Rectified Linear Unit (ReLU) activation function connected to a final layer composed of 8 neurons and a pair for each line and column coordinates of the 4 desired points.

**Figure 5:**
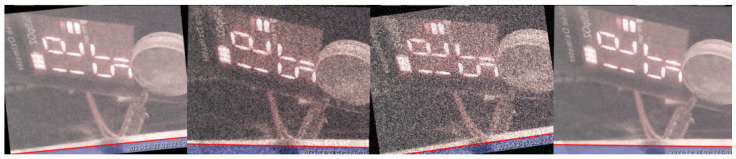
Samples generated using image transformations.

In the training procedure, the original weights of VGG16 were not modified; only the weights of the input and output layers were modified. From all 22 images, 16 were used for training and 6 for the validation of our model. The images in each group were selected at random. To avoid overfitting of our model and increase the noise and motion robustness of VGG16, multiple random image transformations, such as rotation, Gaussian noise addition, contrast, and shear, were applied at random during the training Figure 5). These transformations were performed at each epoch by selecting random samples and applying the image processing techniques on them. This procedure allows generating multiple training samples using only one image. A normalization procedure was applied on the image pixels’ intensities, dividing them by 255 (maximum intensity of the images with 8 bit colour resolution) to have a maximum value of 1.

The Mean-Squared Error (MSE) loss metric was used in the training process, and the Adam optimizer was applied. To keep the best weights and avoid overfitting, a checkpoint function was created, which saves the model with the lowest validation MSE. The model was trained for 150 epochs with 32 randomly generated images in each epoch. The code was developed and trained using the free version of the Google Colab virtual machine (the training was performed with a GPU) using Python and Tensorflow [24].

**Figure 6:**
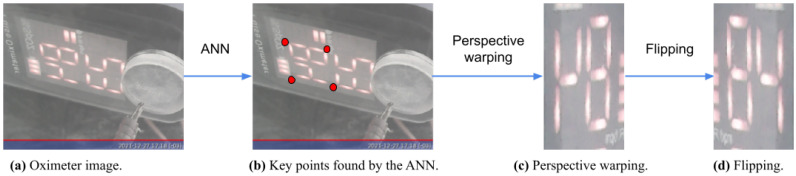
Processing steps for the oximeter display images. (**a**) The oximeter image taken by the camera; (**b**) the ANN output points marked in red; (**c**) the resulting warping procedure given the VGG16 points; (**d**) flipping the image to generate the final image.

Tensorflow lite was used to convert the model into a 32 bit version, allowing the IPMA to be embedded on a Raspberry board. Then, it was used to find the key points in the images. A perspective transformation was performed to align and segment the SpO2 reading, and finally, a vertical flip was applied. The full procedure of the image alignment applied to the oximeter is presented in Figure 6.

#### 3.1.2. Character Recognition

**Figure 7:**
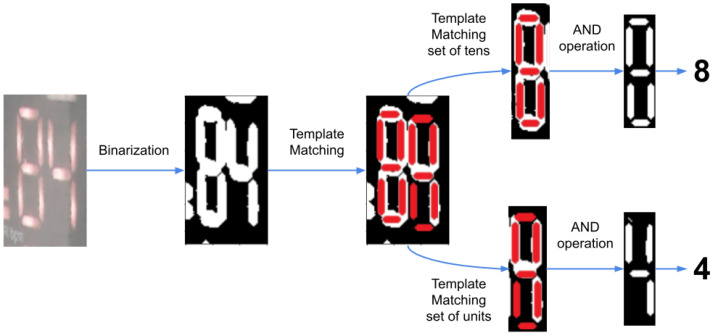
Diagram of the OCR recognition algorithm using the aligned image (the oximeter display in this example) to search for key pixels applying template matching and return the displayed value in text format.

Once the images are aligned by using VGG16, the OCR algorithm takes the images and binarises them, and a template matching algorithm is used to locate the segments of the 7-segment digits in the image. This is performed in two parts, first finding the position of the full number, then performing the template matching on each digit. Afterwards, the algorithm checks if the segment is activated or not and then compares it with a 7-segment display table, thus determining which digit, from 0 to 9, is being displayed. This algorithm has been demonstrated to be a 100% efficient. Thus, once the digits are recognized, it is possible to know the respective number shown in the display, knowing the position of each digit. Figure 7 shows the diagram of the OCR recognition system.

### 3.2. COVID-19 Inference

**Figure 8:**
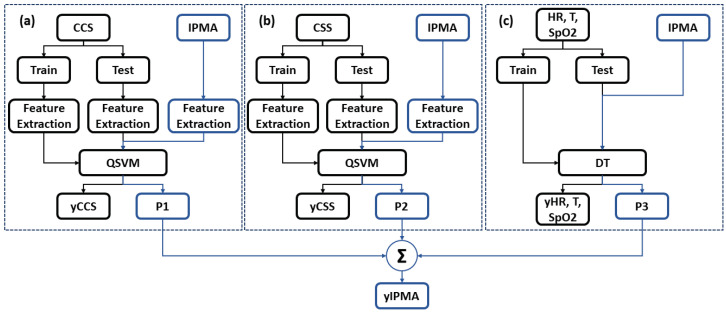
Block diagram of the machine learning algorithms used in this study. (**a**) Cough signal; (**b**) speech signal; (**c**) heart rate, body temperature, and SpO2.

In our research, we used cough and audio sounds (from the CCS and CSS of the Cambridge COVID-19 Sound database [22]), in addition to the data of body temperature, heart rate, and SpO2 from [17], to train our machine learning algorithms (Quadratic kernel-free non-linear Support Vector Machine (QSVM) and Decision Tree (DT) with Gini’s diversity index to split the data), and then, we applied them to the data collected by the IPMA. Figure 8 shows the block diagram for these algorithms.

Prior to any processing, we re-sampled the cough and speech data to a frequency of 16 kHz. For each audio sample, the Mel spectrogram was extracted with a window frame of 25 ms for processing and an overlap of 50%. These spectrograms are computed by extracting the coefficients relative to the compositional frequencies with Short-Time Fourier Transform (STFT), such as done by [31]. Following, these Mel spectrograms are converted to grey-scale, and 512 Local Ternary Pattern (LTP) features are extracted. This feature extraction method is an extension of LBPs, which uses a constant threshold to threshold pixels into three values, i.e., −1, 0, and 1 [32,33]. For heart rate, temperature, and SpO2 signals, the feature vector is composed of their values. The three feature vectors obtained are used to train twodifferent classifier models: for cough and speech, a QSVM is used, whereas for heart rate, body temperature, and SpO2, a DT is used. For each dataset, approximately 70% of the data were used as the training set and 30% for testing. Furthermore, the full training set was analysed in a k-fold cross-validation, with k=10. The test and IPMA’s values’ set were checked using each classifier model, and a score vector of the individual being infected or non-infected was obtained for the IPMA set (P1, P2, and P3). This score is the distance from the sample to the decision boundary. A positive score for a class indicates that the sample is predicted to be in that class. A negative score indicates otherwise. Finally, for the IPMA, the sample scores were summed, and if the highest value was related to the COVID-19 score, the sample was assigned as “infected”.

The data collected by the IPMA from 36 volunteers (from Ecuador and Brazil, shown in Appendix A were used to evaluate the classifiers’ models previously trained with the CCS, CSS, and the database from [17]. The cough signals were preprocessed, and the feature extraction was performed over these three datasets and the IPMA database. Table 2 shows results regarding the Accuracy (ACC) and Area Under the Curve (AUC) obtained for the datasets from the CCS, CSS, and [17], using QSVM for cough and speech and DT for the dataset with body temperature, heart rate, and SpO2. For the IPMA’s data, an ACC of 100% was achieved.

## 4. Evaluation Metrics Applied to the IPMA

The methodology used to evaluate the IPMA is based on its ease of use and if the GUI is engaging for the volunteer. In order to evaluate the IPMA and the GUI, two scales were used here, which were the System Usability Scale (SUS) and the Post Study System Usability Questionnaire (PSSUQ).

The SUS is a methodology to evaluate the usability, effectiveness, and efficiency of a system, which was developed by Brooke in 1986 as a 10-question survey [34]. In the SUS, the volunteer gives a score for each question, ranging from 1 to 5. The value 1 means the volunteer totally disagrees with the sentence being asked, whereas 5 means the individual totally agrees. The SUS sentences are shown below [35]:

1. I think that I would like to use this system frequently.
2. I found the system unnecessarily complex.
3. I thought the system was easy to use.
4. I think that I would need the support of a technical person to be able to use this system.
5. I found the various functions in this system were well integrated.
6. I thought there was too much inconsistency in this system.
7. I would imagine that most people would learn to use this system very quickly.
8. I found the system very cumbersome to use.
9. I felt very confident using the system.
10. I needed to learn a lot of things before I could get going with this system.

The way the sentences are organized has even questions, with a positive view of the system, and odd questions, with a negative view. There is a similar odd question for each even question, but written in a different way. The Total SUS score (T) is calculated using Equation (1), where *p* is the score of each question. Note that the parity of the question influences its contribution to the total score.

(1) $$T=2.5\left[\left(\sum_{odds}p-1\right)+\left(\sum_{even}5-p\right)\right]$$

Although the SUS scale ranges from 0 to 100, it is not a percentile scale; there is a graph used to correlate the percentile scale with the SUS score, with the average (50%) represented by the score 68 [36]. In addition, according to [37], scores of the SUS above average are associated with “Good” products.

As previously mentioned, another important aspect of usability that should be evaluated is the user experience with the computational application, which is addressed by the PSSUQ. This scale is a 19-element standardized questionnaire, which can be scored from 1 (“I strongly agree”) to 7 (“I totally disagree”), 4 being “neutral”. It is used to evaluate the user experience with computer systems and applications and was developed by IBM in 1988 from a project called System Usability Metrics (SUMS) [36]. It follows a 7-point Likert scale, and the result is the average score of the questions.

An interesting aspect of this scale is that it can be split into three subcategories to evaluate three different aspects of the user experience, which are the System Usefulness (SYSUSE), Information Quality (INFOQUAL), and Interface Quality (INTERQUAL). The SYSUSE is calculated by averaging the results from Questions 1 to 6, whereas the INFOQUAL and INTERQUAL average the results for Questions 7 to 12 and 13 to 15, respectively. Additionally, there is the overall score, which is the average of all questions, including the 16th question.

In the PSSUQ, lower scores mean better evaluations, thus indicating a higher level of usability. The neutral value is 4, whereas values closer to 1 represent better usability and closer to 7 represent worse usability. The sentences in the PSSUQ scale are [38]:

1. Overall, I am satisfied with how easy it is to use this system.
2. It was simple to use this system.
3. I was able to complete the tasks and scenarios quickly using this system.
4. I felt comfortable using this system.
5. It was easy to learn to use this system.
6. I believe I could become productive quickly using this system.
7. The system gave error messages that clearly told me how to fix problems.
8. Whenever I made a mistake using the system, I could recover easily and quickly.
9. The information (such as online help, on-screen messages, and other documentation) provided with this system was clear.
10. It was easy to find the information I needed.
11. The information was effective in helping me complete the tasks and scenarios.
12. The organization of information on the system screens was clear.
13. The interface of this system was pleasant.
14. I liked using the interface of this system.
15. This system has all the functions and capabilities I expect it to have.
16. Overall, I am satisfied with this system.

### Evaluation Protocol

Following [39], testing a large number of participants does not necessarily provide much more information than testing just a few people, since even a few participants are able to find most of the usability issues. According to them, a usability test performed by five users should be enough to detect up to 80% of the potential problems of a product or website. Thus, the probability of a user finding an issue is about 31%. After those five users, the same findings continue to be observed repeatedly without discovering anything new. Thus, based on their study, we selected 18 people for the tests of each IPMA (from Ecuador and Brazil), resulting in a total of 36 individuals.

In the evaluations, the volunteer had to follow the instructions to have his/her biological data collected, and after using the equipment, he/she filled out the SUS and PSSUQ forms. Therefore, it was possible to know their opinion about the usability of the IPMA. Due to the current sanitary restrictions, the equipment was only evaluated with volunteers that did not have ongoing COVID-19 infection, which means they were either recovered or had never had the disease. The evaluation protocol consists of the following steps:

1. Volunteers were given an explanation about the whole process of the use of the equipment.
2. They filled out a questionnaire that included their birthday, gender, and health questions.
3. The system asked the volunteer to open the IPMA’s door and make a 10 s forced cough.
4. Afterwards, the system asked the volunteer to speak a phonetically balanced sentence.
5. Next, the volunteer was informed that the system would take the measurements. The volunteer was asked to place his/her arm properly inside the IPMA. Then, measurements took place after pressing the start button
6. Once the measurements were finished, the IPMA asked the volunteer to remove his/her arm from the IPMA.
7. Further, the system acknowledged the volunteer and started the UV-C disinfection process.
8. Finally, the volunteer was asked to fill out two forms (SUS and PSSUQ).

## 5. Results and Analysis

### 5.1. Evaluation Conducted in Ecuador

In these trials, it was possible to evaluate the equipment’s aspects, including its mechanical features and usability by 18 volunteers. Regarding the age of the individuals, 72.22% (13 individuals) were young adults (15–29 years old) and 27.78% (5 individuals) were adults (30–64 years old). Results for the measurements, as well as the photos of the experiment setup can be viewed in Appendix B.

In terms of the usability of the equipment, the SUS provided a good result (78.19), which was above the average (68). All individuals but one gave scores that reached values better than the average. Regarding the interface evaluation, the scores showed that the GUI was considered to have a high level of usability, as the average was 1.6 (with subscales SYSUSE scoring 1.5, INFOQUAL scoring 1.8, and INTERQUAL scoring 1.4). Appendix B shows the results of the SUS and PSSUQ for Ecuadorian volunteers.

Regarding the concept of the SYSUSE, individuals’ opinions showed that the app was intuitive, even for the first time of use. On the other hand, some individuals, especially those who were older, felt that the need to have a Gmail account was a drawback for the application. In the INFOQUAL subscale, individuals considered the system functionality. For instance, they suggested the IPMA should emit warnings when the network signal is weak or the hand position is not correct. Finally, in the INTERQUAL subscale, the interface was considered friendly for most users; however, they suggested that more graphs would make the system better.

### 5.2. Evaluation Conducted in Brazil

The 18 Brazilian volunteers followed the GUI, which guided them in the process of filling out the form, capturing the audio recordings, and taking the measurements. Afterwards, they were asked to fill out both the SUS and PSSUQ forms to evaluate the IPMA. The detailed results from the IPMA trials are presented in Appendix A.

From the SUS perspective, the equipment scored 81.11 for the Brazilian volunteers, a value considered above average for usability. Five of them (27.78%) scored the equipment below average (68), 2 out of those 5 scores being slightly below average (67.5 and 65). However, the majority of the individuals (13 of them, i.e., 72.22%) considered the equipment quite useful.

The PSSUQ scores showed that the volunteers found the GUI quite useful, with an average score of 2.4 (where the INFOQUAL had a score of 2.8, the SYSUSE had a score of 2.2, and the INTERQUAL had a score of 2.0). This shows, in general, that the Brazilian volunteers also had a good experience with the equipment.

## 6. Conclusions

This work introduced the all-in-one Integrated Portable Medical Assistant (IPMA), which is a piece of equipment that allows biomedical data acquisition from individuals, infected or not with COVID-19, through sounds of coughing and three biomedical signals (heart rate, oxygen saturation level, and body temperature) acquired from medically certified devices. The values of these biomedical data were obtained through their displays’ readings by cameras and using a 100% efficient VGG16 ANN. All these signals collected from the individual by the IPMA feed pre-trained machine learning algorithms (which achieved approximately ACC = 88.0% and AUC = 0.85 and ACC = 99% and AUC = 0.94, respectively, using QSVM and DT) to allow inferring possible COVID-19 infection, with 100% accuracy, thus indicating to the individual when it is time to seek medical care. It is important to say that although the data collected by the IPMA were all from individuals without COVID-19 infection, our machine learning algorithms showed a good performance to infer COVID-19 from different databases.

Regarding the evaluation from the individuals who used the IPMA, it was considered successful, since it achieved an average score over 68 on the SUS, which means the equipment was considered “above average” (or, in other words, good equipment). Additionally, the PSSUQ presented low scores for both IPMA versions, which means high overall usability.

It is worth mentioning that the IPMA has great advantages as it is a non-invasive, real-time, any-time equipment for large-scale data acquisition for screening and may be used for daily screening of students, workers, and people in public places, such as schools, jobs, and public transportation, to quickly alert that there are group outbreaks. Furthermore, due to its portability, it is suitable to be used in hospitals, in clinics, or at home. Additionally, the IPMA was designed to be user-friendly, with a comprehensive GUI, and safe, since it uses UV-C light to disinfect it.

With the widespread use of the IPMA and collection of data, a new database is being created, which will be quite useful for new studies about alternative parameters to infer COVID-19 infection and other respiratory diseases, mainly because, more than two years after its outbreak, COVID-19 is still strongly threatening the world with its new variants. Mobile applications (apps) were also developed here to allow the compilation of the main physiological signals captured by the equipment and, then, visualize them in mobile phones.

The IPMA was also proven to be functional, and the evaluations conducted with individuals showed that the measurements can be performed easily, while the results can be stored for further analysis and for machine learning training. As future works, we expect to use this equipment to evaluate other respiratory diseases, such as cold, flu, or pneumonia, by training the machine learning with more data.

Regarding a comparison between the IPMA with other equipment with the same purpose, this was not possible, since, as far as we know, there is no other equipment that tries to perform data acquisition from medically certified devices, create a database, and apply machine learning algorithms in the same way as we did here. Additionally, we tested our equipment with known scales to evaluate usability and user interface quality, in order to validate our research.

The limitations of our work are mainly the need for an AC outlet near the equipment to power the UV-C lamps, as well as the need for batteries for each medical device, since we could not power them directly with the main IPMA battery due to the risk of losing their medical certification. Additionally, the structure became bigger because of the need for linear actuators and cameras, as we could not obtain the measurement signals directly from the electronic board of the medically certified devices, to avoid losing their medical certification.

As future works, we plan to perform more trials in different geographical areas to collect more data and verify the usability with people of different regions. Furthermore, we will add some improvements to the user interface to make it more user-friendly and to improve the visualization in the mobile application. Finally, tests will be performed at public healthcare institutions to evaluate the use of the device.

## Figures and Tables

**Table 1:** Features of each version of the IPMA.

	Version A	Version B
Size	22 × 28.5 × 49.6 (cm${}^{3}$)	30 × 70 × 70 (cm${}^{3}$)
Oximeter	Shenzen IMDK (C101A3)	Hunan Accurate Bio-Medical (FS10K)
Thermometer	Easy East (model IR200)	Bioland (E122)
Microphone	Knup (KP-911)	Knup (KP-911)
Disinfection	External UVC	Embedded UVC

**Table 2:** Results from the CCS, CSS, and [17] datasets.

	CCS—Using QSVM	CSS—Using QSVM	[17]—Using DT
ACC (%)	87.98	70.32	98.62
AUC	0.85	0.66	0.94

**Figure A1:**
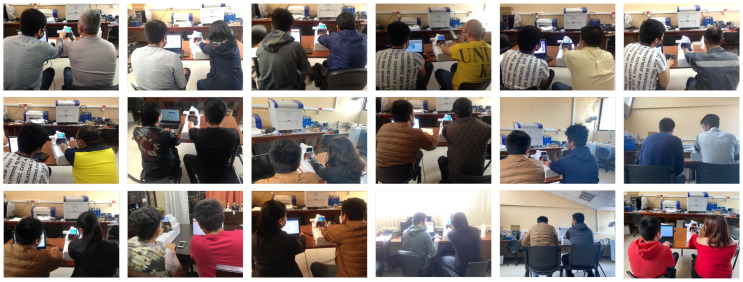
Ecuadorian volunteers taking the tests with the IPMA.

**Figure A2:**
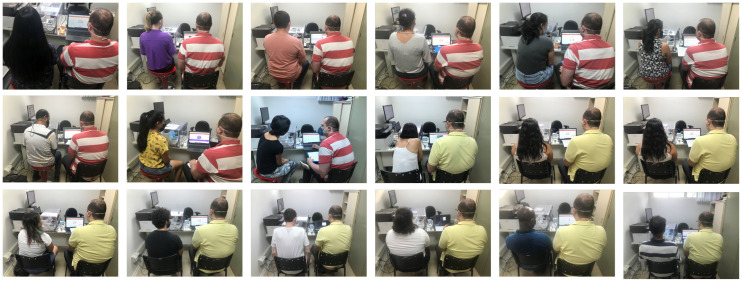
Brazilian volunteers taking the tests with the IPMA.

## Data Availability

Not applicable.

## Appendix A. Measurements Conducted with the IPMA

Figure A1 and Figure A2 show mosaics with photos of the Ecuadorian and Brazilian volunteers taking the tests.

## Appendix B. Measurements Conducted with the IPMA

### Appendix B.1. Measurements

Table A1 shows the data of oxygen saturation, heart rate, and body temperature, taken with the IPMA, in addition to the information of the birth date, gender, and whether the volunteer is infected or not with COVID-19. In this table, SpO2 is the oxygen saturation level (in percentage), HR is the Heart Rate (in bpm—beats per minute), Temp is the body temperature (in degrees Celsius), and SBP and DBP are, respectively, Systolic Blood Pressure and Diastolic Blood Pressure, measured in millimetres of mercury (mmHg). In the column Gender, M stands for Male and F for Female. In the column COVID, R stands for Recovered and N for Negative, RT-PCR refers to the Real-Time Polymerase Chain Reaction, NS means No Symptoms, ME means Medical Evaluation, and PE means Personal Evaluation. The columns Onset and Finish refer to the date of the beginning of the symptoms and the date that the symptoms ended, respectively.

**Table A1:** Measurements and volunteers’ information taken during the IPMA’s evaluation by Ecuadorian volunteers.

ID	Gender	Birth Date	COVID	Onset	Finish	SpO2	HR	Temp	SBP	DBP
1	M	06/29/97	R (RT-PCR)	08/18/21	08/21/21	98	93	36.4	45	81
2	M	07/19/99	R (RT-PCR)	08/09/21	08/23/21	94	76	36.2	63	110
3	M	11/26/71	N (RT-PCR)	**-**	**-**	93	68	36.1	70	89
4	F	31/1/1999	N (NS)	**-**	**-**	81	83	35.2	71	111
5	M	04/21/97	R (RT-PCR)	06/15/21	07/15/21	93	73	35.4	49	107
6	M	01/18/95	R (RT-PCR)	05/03/21	05/10/21	98	79	36.0	70	102
7	M	07/08/97	R (RT-PCR)	06/08/21	06/22/21	92	68	35.9	86	117
8	F	12/19/98	R (RT-PCR)	01/10/22	01/31/22	92	73	36.2	65	111
9	F	11/02/99	N (NS)	-	-	89	63	36.0	60	91
10	M	06/18/98	N (NS)	-	-	96	63	36.0	66	98
11	M	01/01/74	N (NS)	-	-	84	77	36.2	52	113
12	M	10/14/97	N (NS)	-	-	96	74	36.2	69	104
13	F	01/07/84	N (NS)	-	-	96	70	36.2	71	102
14	M	02/07/74	N (NS)	01/03/22	01/12/22	95	64	36.1	79	119
15	M	07/13/78	N (NS)	-	-	99	75	36.1	83	117
16	F	01/06/03	R (RT-PCR)	-	-	94	86	35.9	46	85
17	M	07/06/02	N (NS)	-	-	98	88	36.1	43	108
18	M	12/16/03	N (NS)	-	-	91	76	36.3	60	114
Avg	-	-	-	-	-	93.28	74.94	36.03	63.78	104.39

**Table A2:** Measurements and volunteers’ information taken during the IPMA’s evaluation by Brazilian volunteers.

ID	Gender	Birth Date	COVID	Onset	Finish	SpO2	HR	Temp	SBP	DBP
1	F	01/07/1984	N (QT)	-	-	98	72	36.8	98	66
2	F	04/09/1993	R (PE)	27/12/2020	04/01/2021	97	98	36.3	116	79
3	M	05/29/1989	R (PE)	27/12/2020	04/01/2021	98	73	36.5	120	71
4	F	07/19/1985	N (NS)	-	-	97	76	36.9	132	81
5	F	08/09/1981	N (NS)	-	-	98	67	36.5	131	81
6	M	06/04/1973	N (RT-PCR)	-	-	97	68	36.5	106	71
7	F	09/29/1994	N (NS)	-	-	98	78	36.3	119	69
8	F	05/14/1998	N (NS)	-	-	98	82	36.7	112	74
9	F	12/01/1994	N (ME)	-	-	99	94	36.4	118	63
10	F	05/23/1974	N (RT-PCR)	-	-	98	74	36.6	115	105
11	F	01/26/1971	N (ME)	-	-	96	91	36.2	114	52
12	F	11/11/1991	N (ME)	-	-	98	89	36.3	110	58
13	F	01/14/1989	N (NS)	-	-	99	101	36.3	143	95
14	M	12/15/1989	N (RT-PCR)	-	-	96	63	36.3	96	49
15	M	10/04/1991	N (NS)	-	-	95	86	36.1	117	70
16	F	07/09/1989	N (NS)	-	-	97	104	36.3	135	97
17	M	09/12/1981	N (ME)	-	-	97	71	36.4	137	80
18	M	06/29/1965	N (RT-PCR)	-	-	95	69	36.4	125	83
Avg	-	-	-	-	-	97.28	80.89	36.43	119.11	74.67

### Appendix B.2. SUS

Table A3 and Table A4 show the SUS scores given by the volunteers who tried the IPMA. The first column shows the volunteers who took the experiments, from 1 to 18; in the second line, the numbers are related to the SUS sentences, and the column “SUS” is the overall score calculated for each volunteer. The last line is the average.

**Table A3:** SUS results for Ecuadorian volunteers.

	Questions										
**ID**	**1**	**2**	**3**	**4**	**5**	**6**	**7**	**8**	**9**	**10**	**SUS**
1	3	2	2	4	2	4	1	4	3	3	65.00
2	3	5	1	4	1	4	1	5	1	5	90.00
3	2	2	1	5	2	5	2	5	1	1	75.00
4	2	4	1	2	1	4	1	5	2	5	82.50
5	3	3	1	5	1	5	1	5	5	5	80.00
6	5	3	1	2	3	2	3	2	1	3	47.50
7	1	5	1	5	1	5	1	5	1	4	97.50
8	1	4	1	5	1	3	1	5	1	1	82.50
9	1	5	1	4	1	4	1	5	1	4	92.50
10	2	4	1	1	2	4	1	5	1	1	70.00
11	1	4	1	5	1	4	1	5	1	5	95.00
12	5	5	1	1	1	3	1	5	4	3	62.50
13	5	1	1	5	1	4	1	5	1	5	77.50
14	2	5	1	1	1	4	1	5	1	5	85.00
15	1	4	1	3	1	4	1	5	1	4	87.50
16	3	4	1	2	3	4	2	5	1	3	70.00
17	3	5	1	5	2	4	2	5	1	5	87.50
18	2	4	2	2	4	2	2	5	1	2	60.00
Avg	2.50	3.83	1.11	3.39	1.61	3.83	1.33	4.78	1.56	3.56	78.19

**Table A4:** SUS results for Brazilian volunteers.

	Questions										
**ID**	**1**	**2**	**3**	**4**	**5**	**6**	**7**	**8**	**9**	**10**	**SUS**
1	4	2	5	3	4	1	5	1	4	1	85.00
2	4	2	4	4	4	1	3	1	4	4	67.50
3	5	2	5	4	5	1	5	1	5	2	87.50
4	5	1	5	1	5	1	5	1	5	1	100.00
5	5	1	5	2	4	2	5	1	5	1	92.50
6	5	1	5	1	4	1	5	1	4	1	95.00
7	5	1	5	5	5	1	4	1	5	1	87.50
8	5	1	5	1	5	1	5	1	5	1	100.00
9	5	1	5	4	3	3	4	3	5	1	75.00
10	3	1	5	5	3	2	5	2	3	3	65.00
11	5	1	5	1	5	1	5	1	5	1	100.00
12	5	2	3	1	4	2	2	2	3	1	72.50
13	3	2	4	2	5	1	5	1	4	1	85.00
14	3	2	4	4	3	4	4	4	3	1	55.00
15	5	1	5	1	5	1	5	1	5	1	100.00
16	3	3	4	5	3	1	2	3	3	2	52.50
17	2	2	3	4	2	4	4	5	2	2	40.00
18	5	1	5	1	5	1	5	1	5	1	100.00
Avg	4.30	1.50	4.60	2.70	4.10	1.60	4.30	1.70	4.20	1.40	81.11

### Appendix B.3. PSSUQ

The tables with the PSSUQ scores that the volunteers gave at the end of the experiment are given below. Table A5 shows the Ecuadorian results for the PSSUQ and Table A6 the Brazilian results. The numbers 1 to 18 in each table represent the volunteers who took the evaluation; in the second line, there is the number of PSSUQ sentences and the scores for each subscale and for the PSSUQ. In this line, S stands for the subscale SYSUSE, F for INFOQUAL, Q for INTERQUAL, and P for PSSUQ (overall score). The last line is the averages.

**Table A5:** PSSUQ results for the Ecuadorian volunteers.

	Sentences																Scores			
**ID**	**1**	**2**	**3**	**4**	**5**	**6**	**7**	**8**	**9**	**10**	**11**	**12**	**13**	**14**	**15**	**16**	**S**	**F**	**Q**	**P**
1	2	1	1	1	2	3	2	2	4	3	2	3	1	2	2	1	1.7	2.8	1.7	2.0
2	1	1	2	3	2	2	1	1	3	1	1	1	1	1	1	2	1.8	1.4	1.0	1.5
3	1	1	2	2	2	1	1	2	3	2	1	1	1	1	1	1	1.5	1.8	1.0	1.4
4	1	1	1	2	2	1	2	2	2	2	1	1	1	1	1	1	1.3	1.6	1.0	1.4
5	1	1	1	1	1	1	1	1	1	1	1	1	1	1	1	1	1.0	1.0	1.0	1.0
6	4	2	3	4	3	1	2	2	3	3	4	4	3	4	5	5	2.8	3.2	4.0	3.3
7	1	1	1	1	1	2	1	1	6	1	1	1	1	1	1	1	1.2	2.0	1.0	1.4
8	1	1	1	1	1	1	1	1	2	2	1	1	1	1	1	1	1.0	1.4	1.0	1.1
9	1	1	1	1	1	1	1	1	6	1	1	1	1	1	1	1	1.0	2.0	1.0	1.3
10	1	1	1	1	1	1	1	1	1	1	1	1	1	1	1	1	1.0	1.0	1.0	1.0
11	1	2	1	1	1	1	1	1	2	1	1	1	1	1	2	2	1.2	1.2	1.3	1.3
12	5	2	7	3	3	2	1	3	3	3	1	1	3	3	1	2	3.7	2.2	2.3	2.7
13	1	1	1	1	1	1	1	1	6	4	1	1	1	4	1	1	1.0	2.6	2.0	1.7
14	1	1	1	2	1	1	1	1	1	1	1	1	1	1	1	2	1.2	1.0	1.0	1.1
15	1	1	1	1	1	1	1	1	1	1	2	2	1	1	1	1	1.0	1.4	1.0	1.1
16	1	1	1	1	1	2	2	4	1	1	1	2	2	2	1	2	1.2	1.8	1.7	1.6
17	1	2	2	3	1	1	1	4	2	1	1	2	1	1	1	1	1.7	2.0	1.0	1.6
18	1	1	1	2	2	3	3	2	2	1	1	3	2	1	1	1	1.7	1.8	1.3	1.7
Avg	1.4	1.3	1.7	1.9	1.7	1.7	1.6	2.1	3.1	2.1	1.8	2.1	2.0	2.2	2.1	2.3	1.5	1.8	1.4	1.6

**Table A6:** PSSUQ results for the Brazilian volunteers.

	Sentences																Scores			
**ID**	**1**	**2**	**3**	**4**	**5**	**6**	**7**	**8**	**9**	**10**	**11**	**12**	**13**	**14**	**15**	**16**	**S**	**F**	**Q**	**P**
19	2	1	3	3	3	2	3	3	4	2	1	2	1	1	1	3	2.3	2.5	1.0	2.2
20	3	3	3	4	2	2	2	2	7	6	2	2	2	3	2	1	2.8	3.5	2.3	2.9
21	1	1	1	1	1	1	1	1	3	1	2	1	1	1	1	1	1.0	1.5	1.0	1.2
22	1	2	1	1	1	1	1	1	1	1	1	1	2	2	1	1	1.2	1.0	1.7	1.2
23	1	1	2	1	1	1	2	2	2	2	2	2	2	2	1	1	1.2	2.0	1.7	1.6
24	2	2	2	1	2	4	1	1	3	3	3	2	2	2	2	3	2.2	2.2	2.0	2.2
25	1	2	1	1	1	1	2	1	4	3	2	1	1	2	1	1	1.2	2.2	1.3	1.6
26	1	1	1	1	1	1	1	1	1	1	1	1	1	1	1	1	1.0	1.0	1.0	1.0
27	3	1	2	1	1	1	4	4	7	6	1	1	1	1	1	1	1.5	3.8	1.0	2.3
28	2	1	2	2	4	2	1	2	6	4	3	2	2	3	2	4	2.2	3.0	2.3	2.6
29	1	1	1	1	1	1	1	1	1	1	1	1	1	1	1	1	1.0	1.0	1.0	1.0
30	6	3	3	3	5	6	4	6	7	7	3	4	3	2	3	7	4.3	5.2	2.7	4.5
31	1	1	1	1	1	1	1	1	4	4	4	1	1	1	2	1	1.0	2.5	1.3	1.6
32	3	4	3	4	4	4	4	3	5	4	5	5	5	4	4	4	3.7	4.3	4.3	4.1
33	2	2	4	4	4	2	2	4	4	4	4	4	2	2	2	2	3.0	3.7	2.0	3.0
34	4	4	4	5	6	4	4	4	6	6	3	3	2	4	3	3	4.5	4.3	3.0	4.1
35	1	1	1	1	1	1	1	1	7	1	1	1	1	1	1	1	1.0	2.0	1.0	1.4
36	4	5	5	4	4	5	5	4	5	4	5	4	5	5	5	4	4.5	4.5	5.0	4.6
Avg	2.2	2.0	2.2	2.2	2.4	2.2	2.2	2.3	4.3	3.3	2.4	2.1	1.9	2.1	1.9	2.2	2.2	2.8	2.0	2.4

## Appendix C. Health-Related Questions (IPMA Form)

The health-related questions from the survey in the trials with the IPMA are:

1. *Did you have any of these symptoms last week? Please, check those you had.* Multiple choices are available in a set of fever, fatigue, throat ache, respiratory difficulty, persistent pain, chest pressure, diarrhoea, cough, other and none of the above.
2. *Do you think you currently have COVID-19?* Single choice in a set containing Yes, No or Recovered answers.
3. *How do you know whether you currently have COVID-19 or not? Please, specify if you made a test.* Single choice in a set containing “I do not think I have COVID-19 now (no test was made)”, “RT-PCR test”, “Quick test”, “Medical evaluation”, “Personal evaluation” and “Other”.
4. *If you checked “Other”, please specify how you know you have or not COVID-19.* The answer here is a text explaining the symptoms the user had last week.
5. *Symptoms onset and finish date* The answer here are the dates of the beginning and end of the symptoms for those who had the disease.

## References

[1] Wang C., Liu Z., Chen Z., Huang X., Xu M., He T., Zhang Z. (2020). The establishment of reference sequence for SARS-CoV-2 and variation analysis. J. Med. Virol..

[2] Corredor-Vargas M., Torezani R., Paneto G., Bastos-Filho T. (2022). Importance of Sequencing the SARS-CoV-2 Genome Using the Nanopore Technique to Understand Its Origin, Evolution and Development of Possible Cures. XXVII Brazilian Congress on Biomedical Engineering.

[3] Sabino E.C., Buss L.F., Carvalho M.P., Prete C.A., Crispim M.A., Fraiji N.A., Pereira R.H., Parag K.V., da Silva Peixoto P., Kraemer M.U. (2021). Resurgence of COVID-19 in Manaus, Brazil, despite high seroprevalence. Lancet.

[4] Resende P.C., Gräf T., Paixão A.C., Appolinario L., Lopes R.S., Mendonça A.C., da Rocha A.S., Motta F.C., Neto L.G., Khouri R. (2021). TA potential SARS-CoV-2 variant of interest (VOI) harboring mutation E484K in the Spike protein was identified within lineage B.1.1.33 circulating in Brazil. Viruses.

[5] Johns Hopkins University. https://coronavirus.jhu.edu/map.html.

[6] Torezani R., Corredor-Vargas M., Ardisson J., Pirovani M., Santos P., Paneto G., Bastos-Filho T. (2022). Molecular dynamics of the COVID-19 pandemic in Espirito Santo (Brazil) and border States. Rev. Inst. Med. Trop. São Paulo.

[7] reliefweb (2022). Two Years into COVID-19 Pandemic, Less Than 10% of People Living in Crisis Are Vaccinated; Just $96 million—Less Than 1% of the Health Budgets of the US, UK, EU and Germany—Is Needed to Vaccinate People in IRC’s Operating Areas. https://reliefweb.int/report/world/two-years-covid-19-pandemic-less-10-people-living-crisis-are-vaccinated-just-96-million.

[8] DynaMed. https://www.dynamed.com/condition/covid-19-novel-coronavirus.

[9] Oliveira B.A., Oliveira L.C., Sabino E.C., Okay T.S. (2020). SARS-CoV-2 and the COVID-19 disease: A mini review on diagnostic methods. Rev. Inst. Med. Trop. São Paulo.

[10] Coelho Y., Lampier L., Valadão C., Caldeira E., Delisle-Rodríguez D., Villa-Parra A.C., Cobos-Maldonado C., Calle-Siguencia J., Urgiles-Ortiz F., Bastos-Filho T. Towards the use of artificial intelligence techniques in biomedical data from an integrated portable medical assistant to infer asymptomatic cases of COVID-19. Proceedings of the Information Technology & Systems.

[11] Oran D.P., Topol E.J. (2020). Prevalence of asymptomatic SARS-CoV-2 infection: A narrative review. Ann. Intern. Med..

[12] Llanos K., Landi C., Yupa F., Vasquez P., Criollo I., Calle-Siguencia J., Urgilés-Ortiz F., Villa-Parra A.C. (2022). Prototype of a Device for the Automatic Measurement of Physiological Signals to Assist the Diagnosis and Monitoring of patients with COVID-19. Ingenius. Rev. Cienc. Y Tecnol..

[13] Topol E. (2020). Is My Cough COVID-19?. https://www.thelancet.com/journals/lancet/article/PIIS0140-6736(20)32589-7/fulltext.

[14] Laguarta J., Hueto F., Subirana B. (2020). COVID-19 Artificial Intelligence Diagnosis Using Only Cough Recordings. IEEE Open J. Eng. Med. Biol..

[15] Symptoms of Coronavirus. https://www.webmd.com/lung/covid-19-symptoms#1.

[16] Mishra T., Wang M., Metwally A.A., Bogu G.K., Brooks A.W., Bahmani A., Alavi A., Celli A., Higgs E., Dagan-Rosenfeld O. (2020). Pre-symptomatic detection of COVID-19 from smartwatch data. Nat. Biomed. Eng..

[17] Shaik H. (2021). Assessing the Infection Status of COVID-19 Patients Using a Wearable Prototype. OpenAire. https://zenodo.org/record/4766192#.Ypis5exBxPY.

[18] Stokes K., Castaldo R., Federici C., Pagliara S., Maccaro A., Cappuccio F., Fico G., Salvatore M., Franzese M., Pecchia L. (2022). The use of artificial intelligence systems in diagnosis of pneumonia via signs and symptoms: A systematic review. Biomed. Signal Process. Control.

[19] Sharma N., Krishnan P., Kumar R., Ramoji S., Chetupalli S.R., Nirmala R., Ghosh P.K., Ganapathy S. (2020). Coswara—A Database of Breathing, Cough, and Voice Sounds for COVID-19 Diagnosis. Proceedings of the Interspeech 2020.

[20] Sharma G., Umapathy K., Krishnan S. (2022). Audio texture analysis of COVID-19 cough, breath, and speech sounds. Biomed. Signal Process. Control.

[21] Hussain S.A., Al Bassam N., Zayegh A., Al Ghawi S. (2022). Prediction and Evaluation of healthy and unhealthy status of COVID-19 patients using wearable device prototype data. MethodsX.

[22] Simonyan K., Zisserman A. (2014). Very Deep Convolutional Networks for Large-Scale Image Recognition. arXiv.

[23] Chollet F. (2015). Keras. https://keras.io.

[24] Abadi M., Agarwal A., Barham P., Brevdo E., Chen Z., Citro C., Corrado G.S., Davis A., Dean J., Devin M. (2015). TensorFlow: Large-Scale Machine Learning on Heterogeneous Systems. tensorflow.org.

[25] Nesselroad J.M., Flacco V.A., Phillips D.M., Kruse J. (1996). Accuracy of automated finger blood pressure devices. Fam. Med..

[26] Kopp J.A. (2021). A Selfie Video Can Measure Your Blood Pressure. https://www.phillyvoice.com/blood-pressure-measure-smartphone-selfies-videos.

[27] Jordao A., Nazare A.C., Sena J., Schwartz W.R. (2019). Human activity recognition based on wearable sensor data: A standardization of the state-of-the-art. arXiv.

[28] Orlandic L., Teijeiro T., Atienza D. (2021). The COUGHVID crowdsourcing dataset, a corpus for the study of large-scale cough analysis algorithms. Sci. Data.

[29] Han J., Xia T., Spathis D., Bondareva E., Brown C., Chauhan J., Dang T., Grammenos A., Hasthanasombat A., Floto A. (2022). Sounds of COVID-19: Exploring realistic performance of audio-based digital testing. NPJ Digit. Med..

[30] Brown C., Chauhan J., Grammenos A., Han J., Hasthanasombat A., Spathis D., Xia T., Cicuta P., Mascolo C. (2020). Exploring Automatic Diagnosis of COVID-19 from Crowdsourced Respiratory Sound Data. Proceedings of the KDD’ 20—26th ACM SIGKDD International Conference on Knowledge Discovery & Data Mining.

[31] Nanni L., Maguolo G., Brahnam S., Paci M. (2021). An ensemble of convolutional neural networks for audio classification. Appl. Sci..

[32] Sharma G., Umapathy K., Krishnan S. (2020). Trends in audio signal feature extraction methods. Appl. Acoust..

[33] Turan C., Lam K.M. (2018). Histogram-based local descriptors for facial expression recognition (FER): A comprehensive study. J. Vis. Commun. Image Represent..

[34] Brooke J. (2013). SUS: A Retrospective. J. Usability Stud..

[35] Brooke J. (1995). SUS: A quick and dirty usability scale. Usability Eval. Ind..

[36] Lewis J.R. (2018). The System Usability Scale: Past, Present, and Future. Int. J. Hum.–Comput. Interact..

[37] Sauro J. (2018). 5 Ways to Interpret a SUS Score. MeasuringU. https://measuringu.com/interpret-sus-score/.

[38] Lewis J.R. (1995). IBM computer usability satisfaction questionnaires: Psychometric evaluation and instructions for use. Int. J. Hum.-Comput. Interact..

[39] Nielsen J., Landauer T.K. A mathematical model of the finding of usability problems. Proceedings of the Conference on Human Factors in Computing Systems—Proceedings.

